# Renal puncture access using a blunt needle: proposal of the blunt puncture concept

**DOI:** 10.1007/s00345-021-03927-8

**Published:** 2022-01-14

**Authors:** Bingbing Hou, Mingquan Wang, Ziyan Song, Qiushi He, Zongyao Hao

**Affiliations:** 1 Department of Urology, The First Affiliated Hospital of Anhui Medical University, Hefei, China; 2 Institute of Urology, Anhui Medical University, Hefei, China; 3 Anhui Province Key Laboratory of Genitourinary Diseases, Anhui Medical University, Hefei, China; 4 Department of Radiology, The First Affiliated Hospital of Anhui Medical University, Hefei, China

**Keywords:** Needles, Nephrostomy, Percutaneous, Renal artery, Haemorrhage, Animal

## Abstract

**Purpose:**

Severe haemorrhage in percutaneous nephrolithotomy (PCNL) is an alarming event, and preventing injury to renal major vessels is a challenge. We evaluated the efficiency of a blunt needle in renal puncture procedures.

**Methods:**

We first retrospectively reviewed the embolization images of post-PCNL patients to analyse the types of arteries injured, which were considered target arteries. Then, either a blunt needle or a conventional needle was used to directly puncture target arteries in ex vivo porcine kidneys and to establish renal access ex vivo and in vivo. The primary outcome was the incidence of target artery injuries, which were observed by digital subtraction angiography, nephroscopy and 3-dimensional endocasts. The secondary outcome was the rate of excreted fluid per access.

**Results:**

The segmental and interlobar arteries were the most common types of injured arteries that needed to be embolized after PCNL. When these arteries were punctured directly, blunt needles reduced injury (1/20 vs. 16/20; OR 4.750; 95% CI 1.966–11.478; *P* < .001) by 76% compared to injuries induced by conventional needles. Moreover, the blunt needle group also had a significantly lower incidence of these arteries’ injuries ex vivo due to renal puncture and yielded a lower rate of excreted fluid in ex vivo and in vivo renal puncture procedures.

**Conclusion:**

A blunt needle for renal puncture can be effective in reducing injury to renal major arteries and the accompanying haemorrhage. We propose the concept of blunt puncture, which may be a promising method for achieving safe renal access in PCNL.

**Supplementary Information:**

The online version contains supplementary material available at 10.1007/s00345-021-03927-8.

## Introduction

Percutaneous nephrolithotomy (PCNL) is the surgical standard for the treatment of large or complex renal stones [1, 2]. However, urologists’ concerns about severe bleeding complications hinder the application and popularisation of PCNL, especially in medical institutions without a digital subtraction angiography (DSA) device. Regarding ways to reduce the risk of bleeding in PCNL, the current areas of focus are mainly on how to reduce the number and size of access tracts to make the surgery as minimally invasive as possible [3–5] and the use of various technical means to improve the success rate of the centre of renal papillae access puncture [6–8]. Although the current technology and equipment have enabled considerable progress, approximately 1–1.3% of patients still experience blood transfusion [3, 9], and approximately 1% of patients require arterial embolization due to severe bleeding [3].

The effect of puncture needles on bleeding complications of PCNL is often ignored. In fact, the conventional needle tip is sharp and can easily injury renal vessels in the puncture path. Sampaio et al. [10] reported that puncture with a conventional 18-gauge needle through the infundibulum or renal pelvis injured an interlobar or segmental artery in 13.6 to 26.5% of patients. It is known that the major arteries are sufficiently elastic, and blunt operations do not injure them. Moreover, the puncture needle is the only nonblunt instrument used during the establishment of percutaneous renal access.

To study safe renal puncture access, we conducted research from another angle by analysing the types of injured arteries that required embolization to determine which arteries should be treated with the most caution to avoid injury in PCNL. Then, we designed a novel needle with a blunt tip and tested its efficiency and safety in reducing the incidence of these major artery injuries and bleeding during the establishment of renal access, with the aim of achieving safe renal access in PCNL.

## Materials and methods

### Study design and participants

We first retrospectively reviewed the embolization images of patients who experienced severe arterial haemorrhage and required renal arterial embolization among 4533 post-PCNL patients at the First Affiliated Hospital of Anhui Medical University from 2016 to 2020. The types of injured arteries were analysed by an experienced interventional surgeon and were considered target arteries. Then, we evaluated the efficiency of a novel blunt needle for reducing injury to those major arteries and the accompanying haemorrhage due to renal puncture in ex vivo kidney and animal experiments.

Fresh ex vivo kidneys were selected from pigs that were recently slaughtered (Chunran Food Co., Ltd, Hefei, China), and the handling and storage of the kidneys are described in Methods S1. Pigs (25–30 kg) were purchased from Linyou farms. Animal experimental procedures followed the rules of the NIH Guide for the Care and Use of Laboratory and were approved by the Ethics Committee of Anhui Medical University.

Kidneys and pigs were numbered and randomly assigned to the blunt needle group or conventional needle group. The evaluator was blinded to the intervention outcomes. Due to the apparent difference between the two needles, the surgeons were not blinded to the needle used. The primary outcome was the incidence of arterial injuries that may cause severe haemorrhage and require renal arterial embolization. The secondary outcome was the rate of excreted fluid per access.

### Design and selection of a suitable blunt needle

**Fig. 1:**
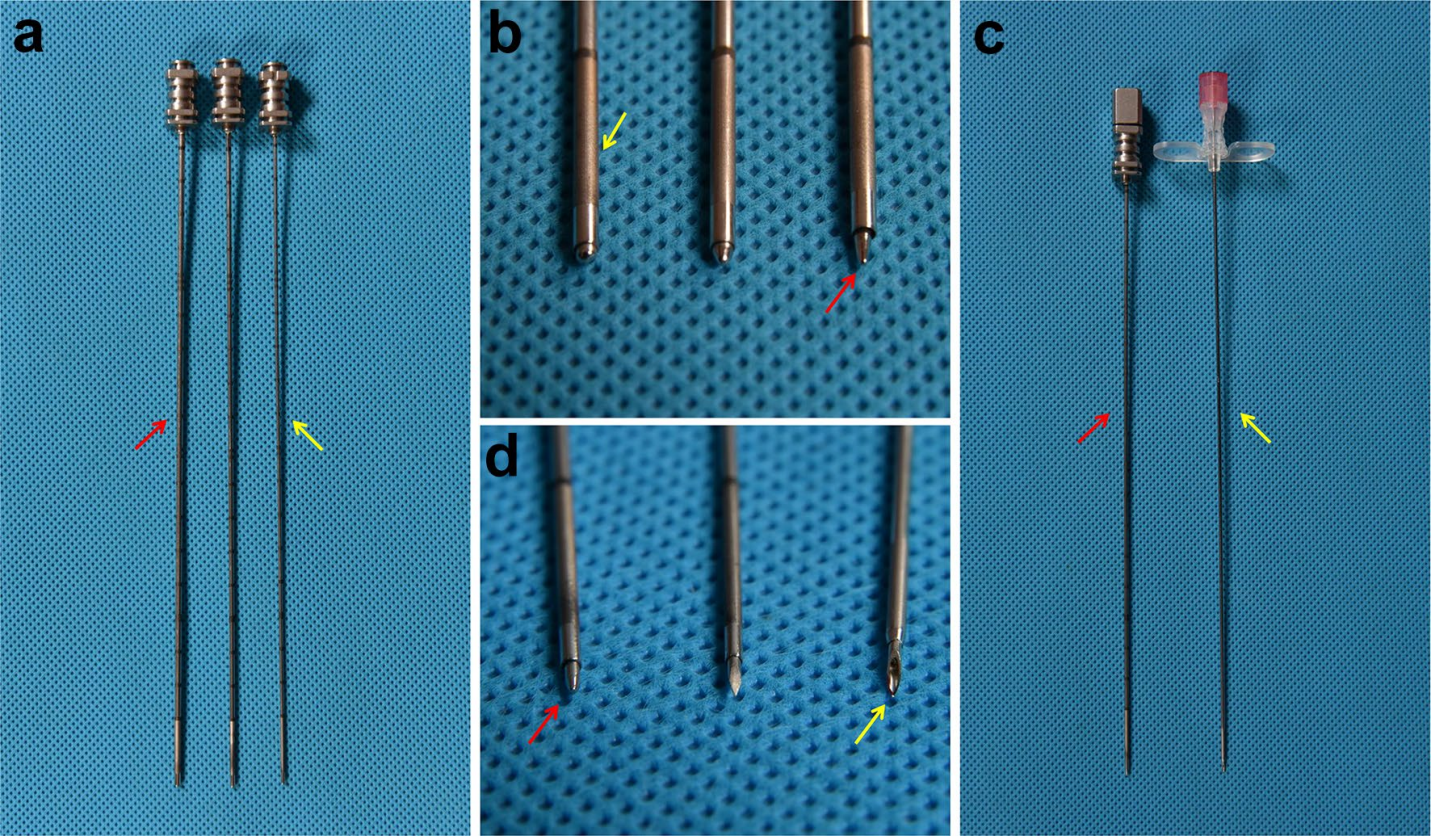
Characteristics of the needles. **a** 16-gauge (red arrow), 17-gauge and 18-gauge (yellow arrow) needle sheaths. **b** Needle core tips with three different degrees of bluntness (red arrow); echo holes (yellow arrow). **c** The selected blunt needle (red arrow) and conventional needle (yellow arrow). **d** The tips of the selected blunt needle (red arrow), auxiliary sharp needle and conventional needle (yellow arrow)

The blunt needle had a needle core with a blunt tip and a needle sheath. The tip of the needle core was similar to an elongated semiellipsoid. The distal end of the needle sheath was designed with dense echo holes, and the needle sheath was marked with a scale line to enable real-time monitoring and allow the depth of needle penetration to be determined by ultrasound. The blunt needle system was obtained from a national practical patent (No: ZL201921738793.1). According to the different thicknesses of the needle sheaths (Fig. 1a) and degrees of bluntness of the needle core tips (Fig. 1b), nine specifications for blunt needles were designed, and a suitable 18-gauge blunt needle was selected (Fig. 1c, d), which could effectively prevent injury to the target arteries and had minimal puncture resistance, similar to a conventional needle (Methods S2).

### Puncture into the target arteries in ex vivo kidneys

**Fig. 2:**
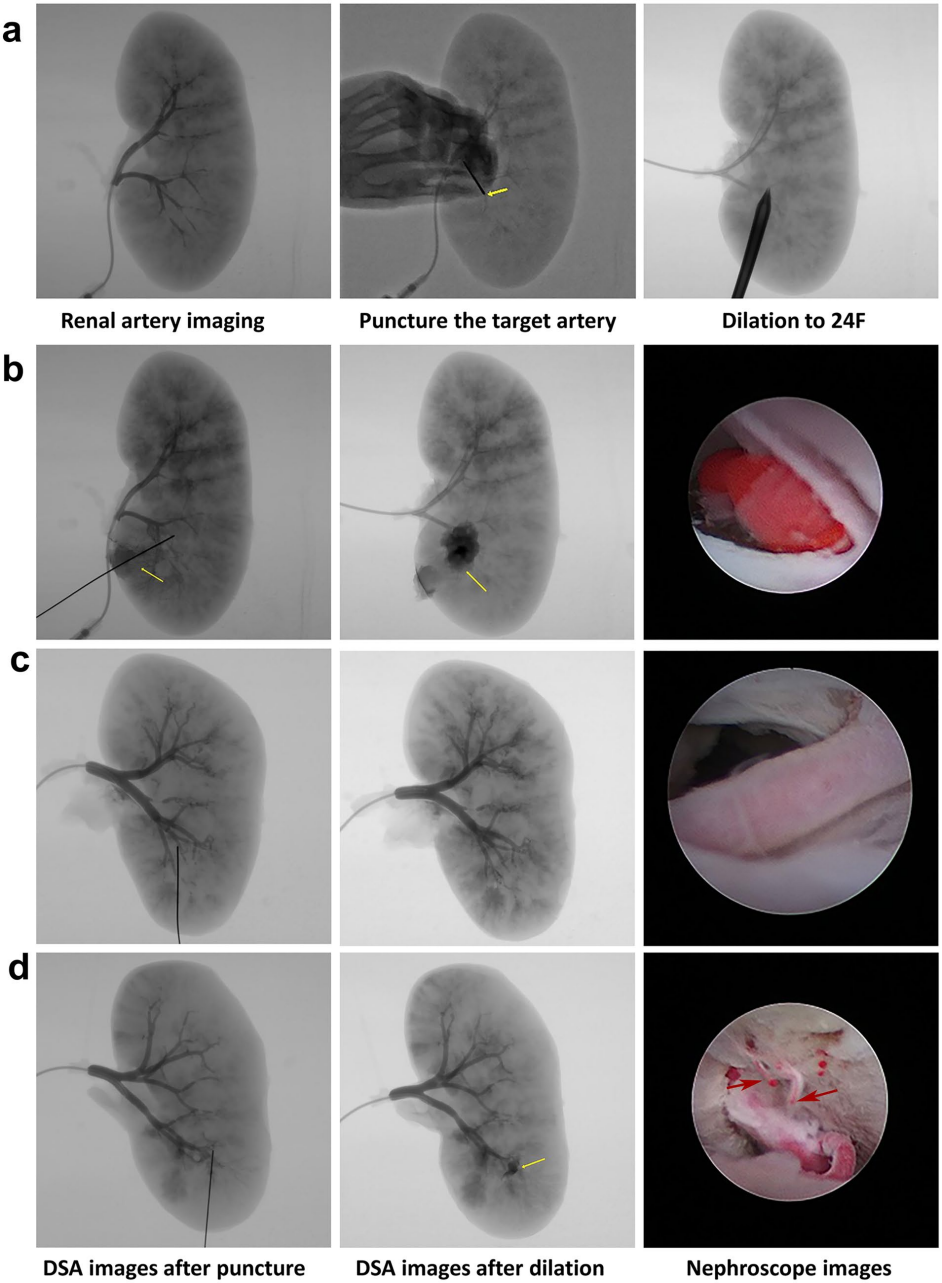
Procedure for puncturing the target artery directly and confirmation of the target artery injury. **a** While DSA showed a normal renal artery, the target artery was directly punctured, and the direction of the puncture was vertical from the ventral area to the dorsal area. **b** There was obvious contrast agent that leaked rapidly from the target artery; after establishment of renal access, more contrast agent leaked, and the injured target artery was found by nephroscopy. **c** No or a small amount contrast agent leaked; after establishment of renal access, there was still no obvious contrast agent that leaked and the target artery was exposed but did not demonstrate any injury under the nephroscope. **d** The target artery was intact, and some small arteries were injured

The renal artery was connected to a high-pressure injection gun, and 50% iohexol was pumped at a rate of 1 ml/s and pressure of 200 cmH_2_O. While using the DSA machine (GE Co., Ltd, Boston, American) to acquire renal angiography images, the target artery was directly punctured (once per kidney) with a blunt needle (*n* = 20) or conventional needle (*n* = 20; 18-gauge, Cook, Bloomington, American; Fig. 1c) under radioscopy (Fig. 2a) by the same interventional surgeon who performed more than 50 interventional operations per year. A guide wire was placed, and then DSA was performed. Renal access was dilated with fascial dilators up to 24 F, and DSA was performed again. Finally, while injecting red resin into the renal artery, injured arteries with red resin overflow in the renal access were observed under a nephroscope (Wolf, Knittlingen, Germany). DSA and nephroscopy images were combined to confirm whether the target artery was injured.

### Establishment of renal accesses in ex vivo kidneys

The artery of the in ex vivo kidney was infused with normal saline (at a pressure of 110 cmH_2_O) to maintain normal arterial pressure. The same experienced surgeon who performed more than 50 PCNLs per year simulated percutaneous renal puncture with a blunt needle (*n* = 60) or conventional needle (*n* = 60) through the upper, middle or lower kidney along the renal Brodel line (3 renal accesses per kidney). The renal access point was dilated with fascial dilators gradually up to 24 F (Fig S1a). Using the F8 catheter with a 1.5 ml air bag to block the opening that provided access in the collection system, the fluid from the renal access was collected and weighed (Fig S1b, c) within 1 min, which was recorded as the rate of excreted fluid per access; this measurement was repeated 3 times, and the average value was taken.

While injecting red resin into the renal artery continuously, we observed the injured arteries with red resin overflow in the renal access under a nephroscope and recorded the location and types of injured arteries. To obtain 3-dimensional endocasts, the renal artery was clamped, and yellow resin was injected into the ureter to fill the collecting system and renal accesses (Methods S3) [10, 11], which allowed us to observe renal accesses and injured arteries. Finally, nephroscopy images and 3-dimensional endocasts were combined to confirm the types of injured arteries [12].

### Establishment of renal accesses in kidneys in vivo

Twenty pigs fasted for 6 h before the operation and were anaesthetized by an injection of pentobarbital sodium (25 mg/kg) through the ear vein, and then placed in a supine position (Fig S1d). Then, a midline abdominal incision was made to separate the bilateral kidneys and ureters (Fig S1e). Artificial hydronephrosis was established, and the same expert surgeon simulated percutaneous renal puncture through the middle kidney along the renal Brodel line (1 renal access per kidney) with a blunt needle (*n* = 20) or conventional needle (*n* = 20). The renal access was dilated gradually up to 24 F (Fig S1f), and blood from the renal access was collected and weighed immediately within 1 min, which was recorded as the rate of excreted fluid per access; this measurement was repeated 3 times.

### Statistical analysis

Data are shown as the median (first quartile, third quartile) or number (proportion). The means of continuous variables were determined using Student’s *t* test or the Mann–Whitney rank sum test, while categorical variables were evaluated using the Chi-square test or Fisher’s exact test. The statistical analysis was compared using SPSS^®^ 23.0. *P* < 0.05 was considered statistically significant.

## Results

### Angiography findings for haemorrhage after PCNL

A total of 54 (1.2%) out of 4533 post-PCNL patients underwent renal arterial embolization. The types of injured arteries included the segmental artery (22.2%, Fig S2a), interlobar artery (66.7%, Fig S2b, c)), arcuate artery (7.4%, Fig S2d) and other unidentified arteries (3.7%). The segmental artery and interlobar artery were the most common types and were considered the target arteries.

### Efficacy of needles in injuries to target arteries

While renal arteriography was performed in ex vivo kidneys, either the segmental artery or interlobar artery was successfully punctured directly (Fig. 2b, c). The overall incidences of injuries to the target arteries (1/20 vs. 16/20; OR 4.750; 95% CI 1.966–11.478; *P* < 0.001), segmental artery (1/10 vs. 9/10; OR 9.000; 95% CI 1.386–58.443; *P* < 0.001), and interlobar artery (0/10 vs. 7/10; OR 3.338; 95% CI 1.293–8.591; *P* = 0.003) were all significantly lower in the blunt needle group than in the conventional needle group. For some cases in which the target artery was not injured by direct puncture with a needle, after establishing 24 F renal access, a small or moderate amount of contrast agent was leaked under DSA, and small injured arteries were found under nephroscopy rather than the injured target artery (Fig. 2d). These findings were observed in arteries in both the blunt and conventional needle groups, 4/19 and 1/4, respectively.

The type of artery injured was confirmed in ex vivo renal accesses under nephroscopy (Fig S3a) with the aid of 3-dimensional endocasts (Fig S3b). Consistent with the above results, the segmental and interlobular arteries in the blunt needle group had a significantly lower incidence of injuries than those in conventional needle group (1/60 vs. 15/60; OR 19.667; 95% CI 2.504–154.47; *P* < 0.001).

### Efficacy of needles for haemorrhage in ex vivo and in vivo renal accesses

The blunt needle group yielded a lower rate of excreted fluid (1.94 (0.94, 2.62) vs. 3.965 (2.405, 6.765) g/min, *P* < 0.001) than the conventional needle group after the establishment of ex vivo renal access. Moreover, the blunt needle group also yielded a lower rate of excreted fluid than the conventional needle group (1.865 (1.215, 2.725) vs. 3.875 (2.385, 5.45) g/min, *P* = 0.007) after the establishment of in vivo renal access.

## Discussion

In PCNL, the anatomical basis of the renal artery and collection system is used to select the centre of the renal pyramid rather than the renal column or infundibulum as the puncture site [11–13]. However, precise needle puncture of the renal collecting system is challenging, and deviation of the target puncture path is not uncommon, which may mean that serious bleeding complications occur [14, 15]. Despite the widespread use of PCNL, Melo et al. [16] reported in a retrospective analysis of 1066 patients of whom 4.8% to 14.8% with complete staghorn stones still experienced severe bleeding requiring blood transfusions. In our centre, the renal arterial embolization rate of post-PCNL patients was still approximately 1.2%, which was similar to that reported by Zeng et al. [3].

Although bleeding is affected by many factors, vascular injury along the puncture access path is still one of the most important reasons [12, 17]. The process of establishing renal access mainly includes two steps: puncture with a needle and dilation with fascial dilators. It is not well known in which process is the main cause of vascular injury. In this study, the results showed that puncture with a conventional 18-gauge needle could directly injure the interlobar and segmental arteries. Interestingly, these arteries that were not injured by direct puncture remained uninjured after the establishment of renal access. However, injuries to some small arteries could be found. The findings showed that puncture with a needle, compared with dilation with fascial dilators, was a more critical factor in causing injury to major arteries during the establishment of renal access.

Uncontrollable bleeding that required embolization or even nephrectomy is one of the most serious complications of PCNL [18, 19]. An artery injured by needle puncture must be completely or partially broken after dilation with fascial dilators and may lead to lacerated renal arteries or pseudoaneurysms in the kidney. If a subsequent injury in the renal artery and vein is occurs, it may lead to the formation of arteriovenous fistulas [20, 21]. The blunt needle has a smooth tip, and the tip slides through the major arteries in the puncture path rather than injuring them. In this study, the results showed that the segmental artery and interlobar artery were the most common types of injured arteries that needed to be embolized after PCNL; additionally, the use of a blunt needle to directly puncture these arteries could effectively prevent them from being injured.

The main purpose of using a blunt puncture needle is to offer safe percutaneous renal access with lower morbidity. This study demonstrated that using a blunt needle to establish renal access in ex vivo porcine kidneys was associated with a lower rate of excreted fluid per access and could be an effective way to reduce injury to the major renal artery, or at least to reduce artery injuries that may cause severe haemorrhage requiring renal arterial embolization. In vivo experiment, we focussed on the use of blunt needles for renal puncture under normal physiological conditions, which could also reduce the rate of excreted fluid during the establishment of renal access. Therefore, puncture with a blunt needle could be a safe and effective approach for reducing arterial injury and bleeding in renal puncture procedures.

The concept of a blunt operation has been widely used in various surgical procedures. For example, the separation of blunt instruments during surgery, liposuction needles used during liposuction [22], and laparoscopic trocars for the insertion of peritoneal dialysis catheters [23] result in less vascular damage than sharp procedures. Similarly, puncture with a blunt needle has been shown to be effective in reducing arterial injury and bleeding. The conventional puncture needle is the only nonblunt instrument used during the establishment of percutaneous renal access. Based on these ideas, we provided the first proposal of the concept of blunt puncture, which may represent a paradigm shift in PCNL.

The major strength of this study is that we pointed out the types of injured arteries that required embolization after PCNL and designed a novel blunt needle that could be effective in minimising injury to these arteries and the accompanying haemorrhage that occurs during renal punctures in ex vivo kidney and animal experiments. Then, we proposed the concept of blunt puncture for PCNL. Another strength of this study is that various methods were used to verify the effectiveness of the blunt puncture needle and procedures were performed by the same designated experienced interventional surgeon or urologist, leading to a very reliable comparison.

However, some limitations should be considered. This study was conducted in pig kidneys ex vivo and in vivo, and subsequent experiments in a clinical study are needed to confirm our findings. Another potential limitation is that we did not use ultrasound-guided renal access for ex vivo kidney and animal experiments, with the aim of highlighting the advantages of blunt needles in the fewer experimental models that are required. Furthermore, the role of tract size was not underestimated, and we selected only 24 F, which is the most commonly used.

In summary, we first found that the segmental artery and interlobar artery were the most common types of injured arteries that required embolization in PCNL. Then, the ex vivo kidney and animal experiments demonstrated that a blunt needle for renal puncture can be effective in reducing injury to renal major arteries and the accompanying haemorrhage during the establishment of renal access. Finally, we proposed a novel concept of blunt puncture, which may be a promising, helpful method for achieving safe renal access in PCNL.

## Supplementary Information

Below is the link to the electronic supplementary material.

Supplementary file1 (DOC 43 KB)

Supplementary file2 Fig S1. Procedure for collecting blood from the renal access area ex vivo and in vivo. (a) Establishment of renal access in ex vivo kidney. (b) The F8 catheter with an approximately 1.5 ml air bag was gently pulled to block the access opening in the collection system. (c) The fluid from the outer exit of renal access was collected. (d), (e) and (f) Establishment of renal access in the kidney in vivo (TIF 3422 KB)

Supplementary file3 Fig S2. The types of embolized arteries in post-PCNL patients. (a) An aneurysm was formed and connected to the segmental artery with arteriovenous fistula (red arrow). (b) An aneurysm was connected to the interlobar artery (red arrow). (c) An interlobular artery was ruptured (red arrow). (d) An aneurysm was connected to the arcuate artery (red arrow) (TIF 1157 KB)

Supplementary file4 Fig S3. Confirmation of the types of injured arteries with the aid of 3-dimensional endocasts. (a) Injured arteries in the renal accesses were observed under nephroscopy. (b) Three-dimensional endocasts clearly show the relationship among the collecting system, renal access and renal arteries (TIF 4112 KB)

## Data Availability

Data are available with permission from the corresponding author.

## Abbreviations

PCNL: Percutaneous nephrolithotomy
DSA: Digital subtraction angiography
