# Supplementary material for: Renal puncture access using a blunt needle: proposal of the blunt puncture concept
Source: World J Urol. 2022 Jan 14;40(4):1035–41. doi: 10.1007/s00345-021-03927-8 (PMC8994716; doi:10.1007/s00345-021-03927-8)
Supplement: Supplementary file 1 — Supplementary file1 (DOC 43 KB) [file 345_2021_3927_MOESM1_ESM.doc]

World Journal of Urology:

**SUPPLEMENTARY MATERIALS**

**Renal Puncture Access Using a Blunt Needle: Proposal of the Blunt Puncture Concept**

Bingbing Hou^1, 2, 3, †^, Mingquan Wang^4, †^, Ziyan Song^1, 2, 3^, Qiushi He^1, 2, 3^, and Zongyao Hao^1, 2, 3, *^

1. Department of Urology, the First Affiliated Hospital of Anhui Medical University, Hefei, China;

2. Institute of Urology, Anhui Medical University, Hefei, China;

3. Anhui Province Key Laboratory of Genitourinary Diseases, Anhui Medical University, Hefei, China;

4. Department of Radiology, the First Affiliated Hospital of Anhui Medical University, Hefei, China.

† Equal study contribution.

∗ Correspondence: Zongyao Hao; E-mail: haozongyao @163.com.

Supplementary Methods 1

Supplementary Methods 2

Supplementary Methods 3

Supplementary Table 1

**Methods S1. Handling and storage of kidneys**

Fresh kidneys were obtained from pigs that were just slaughtered and weighed 100 to 140 kg (mean 122). The pedicle vessels and renal pelvis of the pigs were separated as soon as possible, a catheter was inserted into the renal artery, and 0~4°C UW solution containing heparin (5000:1) was continuously perfused until the renal vein outflow of clarifying fluid and the renal cortex turned pallid. Then, all kidneys were stored in an incubator with 0~4°C UW solution.

**Methods S2.** **Selection of a suitable blunt needle**

The same interventional surgeon directly punctured the target artery with each of the nine blunt needles or conventional needles under radioscopy 2 times with each needle; each kidney was punctured only once (using the same methods as described in the “Puncture to Target Arteries” section of the main text). Conventional needles were used to puncture arteries twice; both times the target arteries were injured, but punctures with any of the blunt needles did not injure the target arteries. Therefore, an 18G needle with minimal blunting of needle core tips was selected.

Subsequently, we retrogradely punctured the selected blunt needle or a conventional needle through the centre of the renal pyramid, side of the renal pyramid and renal column under nephroscopy guidance to evaluate the puncture resistance of the two needles. Each needle was used for puncture 20 times at each of three puncture paths in 6 pig kidneys. Puncture resistance was randomly evaluated by the same urologist and was divided into three grades according to the difficulty experienced when the needle penetrated the kidney. The three grades were easy, moderate and difficult. The results showed that there was no significant difference between the selected blunt needle and conventional needle (Supplementary Table 1).

**Methods S3. Acquisition of 3-dimensional endocasts**

Self-curing denture base resin powder and liquid (1:1) were mixed, and a small amount of dibutyl phthalate was added to the mixture; a small amount of pigment was then added. The mixture marked with red pigment was injected into the artery, and continuous and moderate pressure was maintained until the renal cortex swelled and exhibited faintly visible red blotches. Then, the catheter was clamped. Through observation of the renal access, arteries marked in red could be observed under a [nephroscope](javascript:;), and the number and thickness of the injured arteries were recorded. After observation, a mixture marked with a yellow pigment was injected into the ureter until it flowed out from the renal accesses. The kidney specimen was immersed in 1% formaldehyde for 2 days and soaked in 37% hydrochloric acid for approximately 10~14 days. The corroded cast kidney was rinsed with tap water until only the endocast of systems that had been injected was left. This type of preparation allowed us to observe the renal access and injured artery, and the number and thickness of the injured arteries were recorded.

**Table S1.** Puncture through different sites and the associated resistance evaluation.

|  | Puncture resistance of the needles | | | | | | | | | |  |
| --- | --- | --- | --- | --- | --- | --- | --- | --- | --- | --- | --- |
|  | Conventional needle group | | | | |  | Blunt needle group | | | | P value |
|  | | Easy | Moderate | Difficult | Mean ranks |  | Easy | Moderate | Difficult | Mean ranks |  |
| Centre of the renal pyramid | | 19 | 1 | 0 | 20.00 |  | 18 | 2 | 0 | 21.00 | 0.21 |
| Side of the renal pyramid | | 2 | 14 | 4 | 21.80 |  | 3 | 15 | 2 | 19.20 | 0.369 |
| Renal column | | 0 | 3 | 17 | 22.08 |  | 1 | 5 | 14 | 18.93 | 0.24 |
