# Supplementary figures and images for: Renal puncture access using a blunt needle: proposal of the blunt puncture concept
Source: World J Urol. 2022 Jan 14;40(4):1035–41. doi: 10.1007/s00345-021-03927-8 (PMC8994716; doi:10.1007/s00345-021-03927-8)

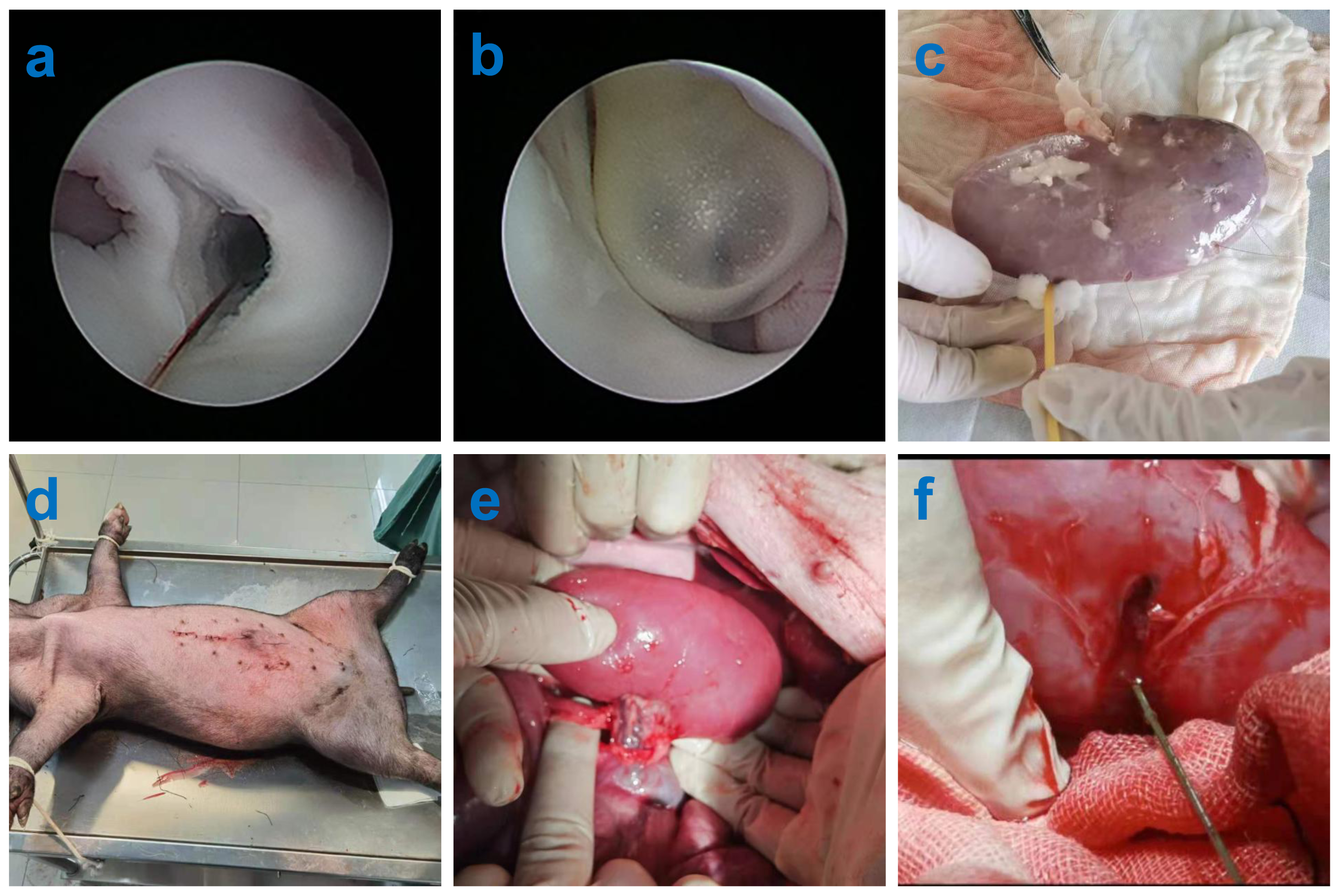

Supplement: Supplementary file 2 — Supplementary file2 Fig S1. Procedure for collecting blood from the renal access area ex vivo and in vivo. (a) Establishment of renal access in ex vivo kidney. (b) The F8 catheter with an approximately 1.5 ml air bag was gently pulled to block the access opening in the collection system. (c) The fluid from the outer exit of renal access was collected. (d), (e) and (f) Establishment of renal access in the kidney in vivo (TIF 3422 KB) [file 345_2021_3927_MOESM2_ESM.tif]

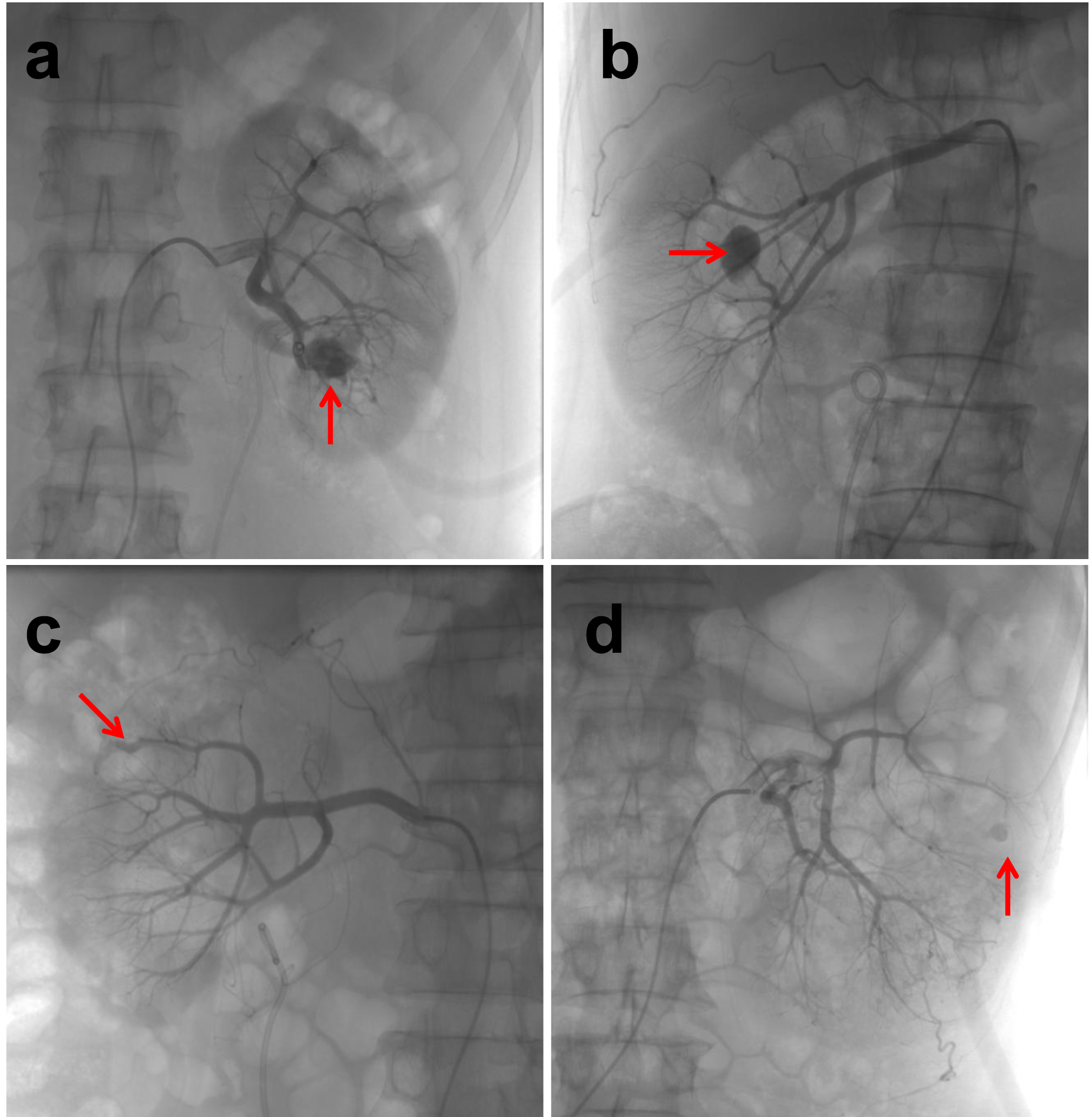

Supplement: Supplementary file 3 — Supplementary file3 Fig S2. The types of embolized arteries in post-PCNL patients. (a) An aneurysm was formed and connected to the segmental artery with arteriovenous fistula (red arrow). (b) An aneurysm was connected to the interlobar artery (red arrow). (c) An interlobular artery was ruptured (red arrow). (d) An aneurysm was connected to the arcuate artery (red arrow) (TIF 1157 KB) [file 345_2021_3927_MOESM3_ESM.tif]

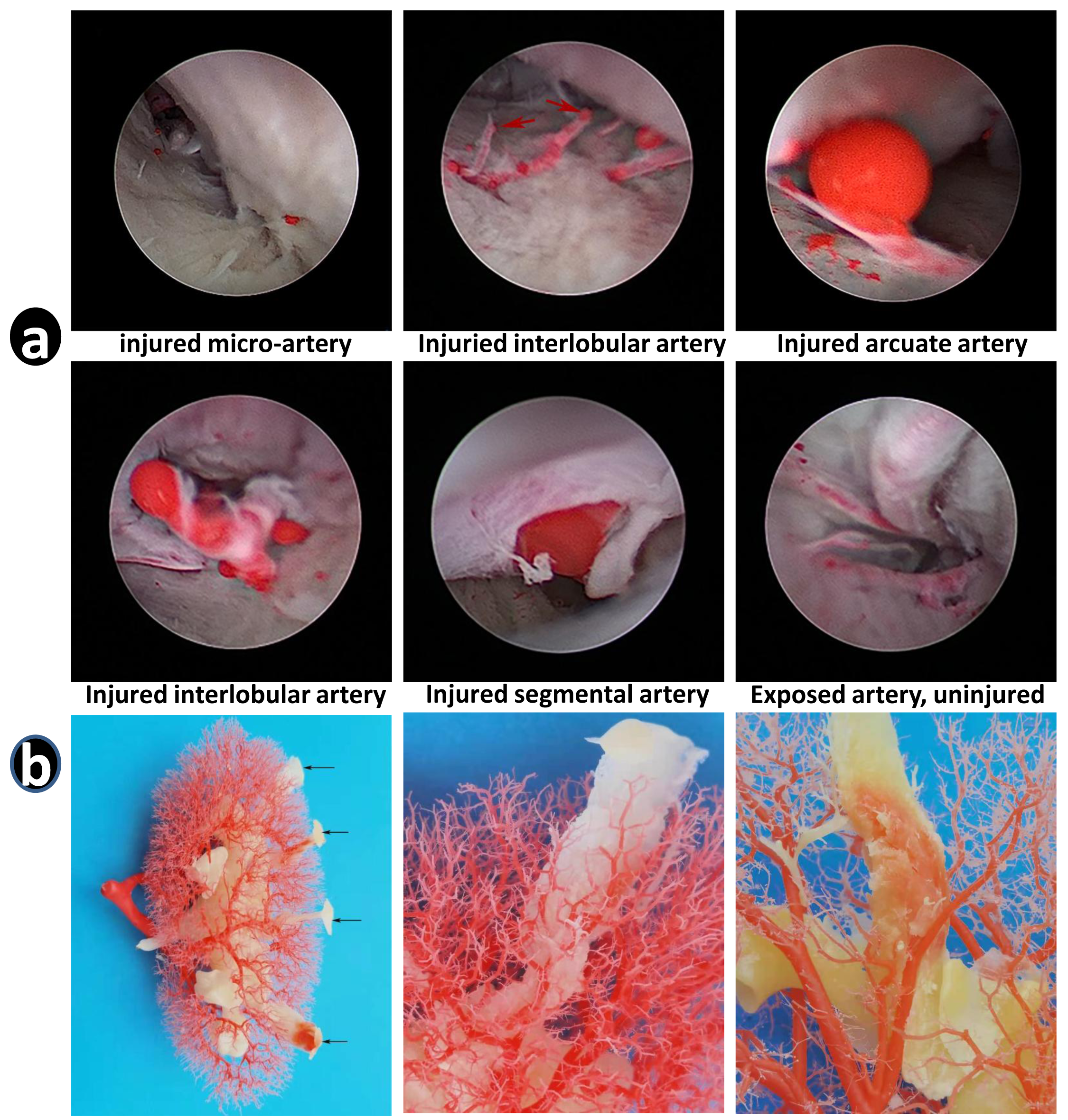

Supplement: Supplementary file 4 — Supplementary file4 Fig S3. Confirmation of the types of injured arteries with the aid of 3-dimensional endocasts. (a) Injured arteries in the renal accesses were observed under nephroscopy. (b) Three-dimensional endocasts clearly show the relationship among the collecting system, renal access and renal arteries (TIF 4112 KB) [file 345_2021_3927_MOESM4_ESM.tif]
